# Anesthetic management in a patient with giant growing teratoma syndrome: a case report

**DOI:** 10.1186/1752-1947-8-32

**Published:** 2014-01-27

**Authors:** Nobuko Ohashi, Hidekazu Imai, Toshiyuki Tobita, Hideaki Ishii, Hiroshi Baba

**Affiliations:** 1 Department of Anesthesiology, Niigata University Medical and Dental Hospital, 1-754, Asahimachi-dori, Chuo-ku, Niigata City 951-8520, Japan

**Keywords:** Anesthesia, Growing teratoma syndrome, Re-expansion pulmonary edema, Respiratory failure, Solid mass

## Abstract

**Introduction:**

Growing teratoma syndrome is a rare occurrence with an ovarian tumor. Anesthesia has been reported to be difficult in cases of growing teratoma syndrome of the cystic type due to the pressure exerted by the tumor. However, there have been no similar reports with the solid mass type. Here, we report our experience of anesthesia in a case of growing teratoma syndrome of the solid type.

**Case presentation:**

The patient was a 30-year-old Japanese woman who had been diagnosed with an ovarian immature teratoma at age 12 and had undergone surgery and chemotherapy. However, she dropped out of treatment. She presented to our hospital with a 40cm giant solid mass and severe respiratory failure, and was scheduled for an operation. We determined that we could not obtain a sufficient tidal volume without spontaneous respiration. Therefore, we chose to perform awake intubation and not to use a muscle relaxant before the operation. At the start of the operation, when muscle relaxant was first administered, we could not obtain a sufficient tidal volume. An abdominal midline incision was performed immediately and her tidal volume recovered. Her resected tumor weighed 10.5kg. After removal of her tumor, her tidal volume was maintained at a level consistent with that under spontaneous respiration to avoid occurrence of re-expansion pulmonary edema.

**Conclusions:**

We performed successful anesthetic management of a case of growing teratoma syndrome with a giant abdominal tumor. Respiratory management was achieved by avoiding use of a muscle relaxant before the operation to maintain spontaneous respiration and by maintaining a relatively low tidal volume, similar to that during spontaneous respiration preoperatively, after removal of the tumor to prevent re-expansion pulmonary edema.

## Introduction

Growing teratoma syndrome (GTS) was first reported by Logothetis *et al.* in 1982 [1]. This syndrome often occurs in testicular nonseminomatous germ cell tumors and is rare in patients with ovarian germ cell tumors. GTS is defined by three criteria: increased metastasis during or after chemotherapy for germ cell tumor, normalization of tumor markers, and the presence of a histologically mature teratoma without malignant elements. Anesthetic management during surgical resection of a giant ovarian tumor of the cystic type has respiratory and circulatory risks in cases of GTS. However, there are no similar reports for resection of solid mass type giant ovarian tumors in cases of GTS. Here, we describe our experience with anesthetic management in a patient with GTS with a solid mass type giant teratoma.

## Case presentation

A 30-year-old Japanese woman (height 161cm, weight 57kg) was diagnosed with an ovarian germ cell tumor at age 12 and underwent total hysterectomy and bilateral oophorectomy. The histological diagnosis was an immature teratoma with metastasis to her lymph nodes. She underwent chemotherapy for a short time after the surgery, but then dropped out of treatment and did not receive further therapy. She gradually developed a recurrent abdominal mass and remained under observation. At age 30, she presented to our hospital requiring an operation, and tumor extirpation was scheduled.

**Figure 1:**
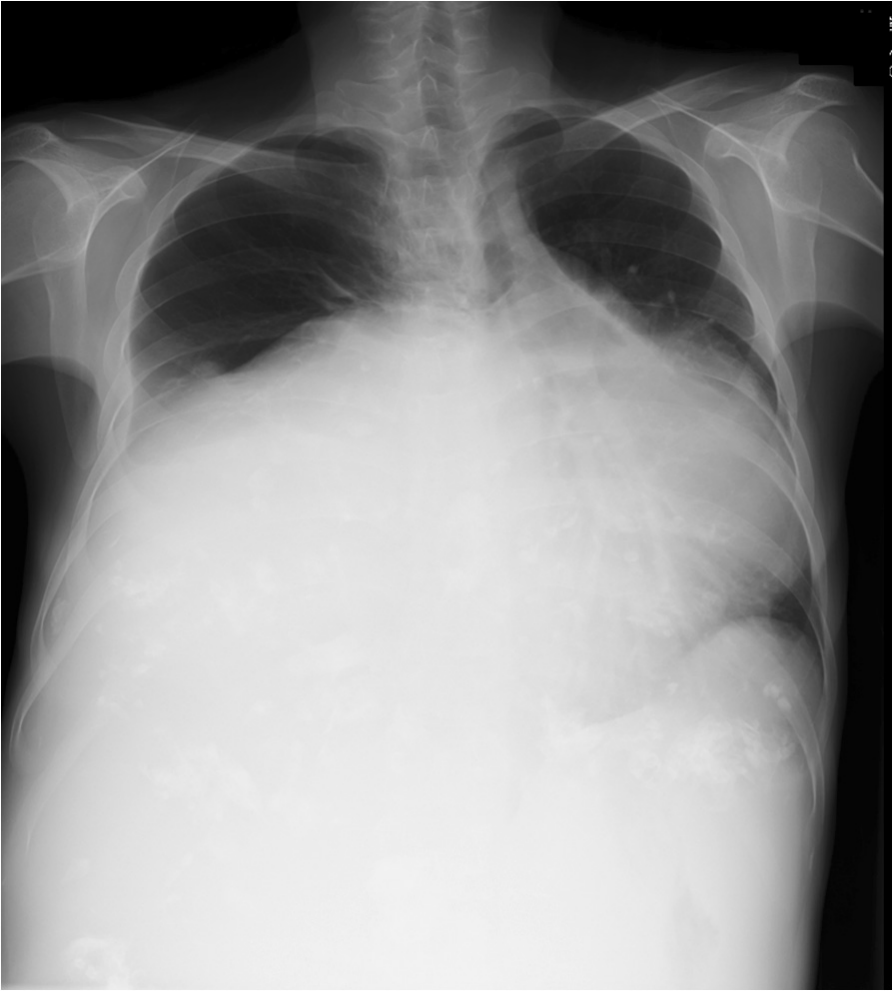
Preoperative chest X-ray showing bilateral diaphragmatic elevation because of the tumor.

**Figure 2:**
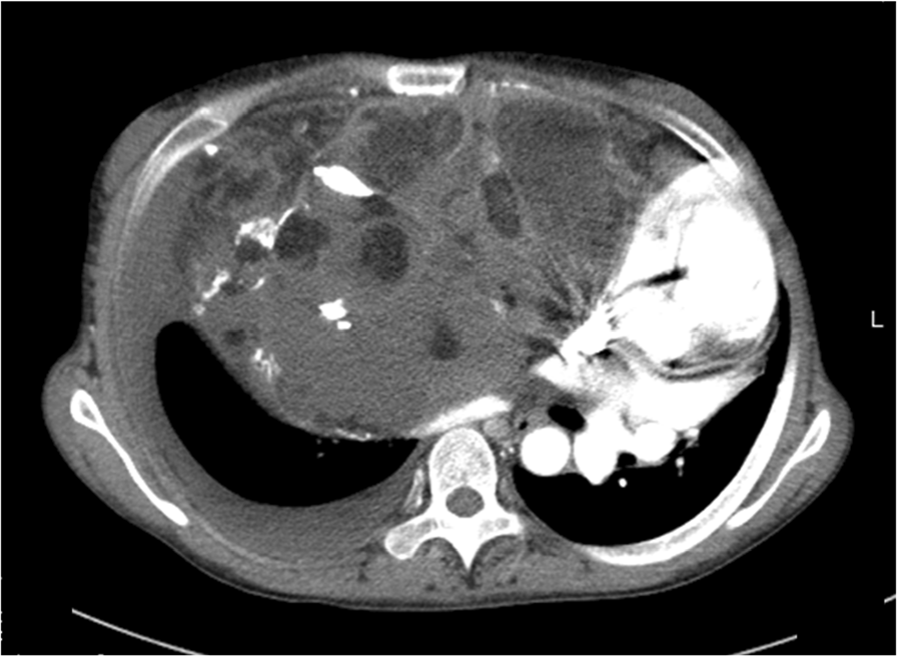
Preoperative computed tomography scan of the abdomen showing a giant solid mass.

A physical examination revealed a huge abdominal mass and a chest X-ray revealed bilateral diaphragmatic elevation. She could not adopt a supine position because of the tumor causing respiratory distress (Figure 1). A computed tomography scan showed a 40×40cm mass (Figure 2). Her vital signs were blood pressure 100/70mmHg, heart rate 100 beats per minute, and pulse oximeter oxygen saturation (SpO_2_) 88 to 90%. Laboratory tests revealed an increased fibrin degradation product of 15.3μg/mL and D-dimer of 2.6μg/mL. Pulmonary function tests showed a low vital capacity (VC) based on the predicted value (%VC 37.7%) and arterial blood gas analysis revealed hypoxia (partial pressure of carbon dioxide, PaCO_2_, 44.6mmHg; partial pressure of oxygen, PaO_2_, 56.2mmHg while breathing room air).

Prior to induction of general anesthesia, an arterial catheter (JELCO I.V. Catheter®; Smith Medical Japan Ltd., Tokyo, Japan) was inserted into her radial artery and a central venous catheter (PreSep Oximetry Catheter®; Edwards Lifesciences, Irvine, CA, USA) was inserted in her left internal jugular vein. Furthermore, percutaneous sheaths (5 Fr Radifocus Introducer®; Terumo Corporation, Tokyo, Japan) were inserted in her femoral artery and vein to use for percutaneous cardiopulmonary support (PCPS) as necessary. Radial arterial pressure, electrocardiogram, capnography, SpO_2_, and cerebral regional oxygen saturation (INVOS 5100®; Somanetics, Troy, MI, USA) were continuously monitored throughout the surgery. After the percutaneous sheaths were inserted, lidocaine 4% was sprayed on her upper airway, while midazolam 1mg and fentanyl 50μg were given intravenously and awake fiberoptic intubation was performed in the Semi-Fowler’s position. We did not use muscle relaxant before the operation because we were concerned about ventilation if spontaneous respiration was lost. During intubation, her tidal volume (TV) decreased slightly because of weakness of spontaneous respiration, but SpO_2_ was maintained and intubation was performed uneventfully. After intubation, midazolam 4mg was administered and anesthesia was maintained with 1.5% sevoflurane. Her TV with spontaneous respiration controlled by pressure support ventilation and positive end-expiratory pressure (PEEP) of 5cmH_2_O was kept between 250 and 350mL.

After surgeons completed preparation for the operation and the surgical site was disinfected, a muscle relaxant (rocuronium 50mg), fentanyl 200μg and 0.3μg/kg/minute remifentanil were administered. Spontaneous respiration was lost and we were unable to obtain a sufficient TV with less than the 20cmH_2_O airway pressure under pressure controlled ventilation or manual ventilation. However, an abdominal midline incision was performed by the surgeon and her TV gradually increased. Her TV further increased and SpO_2_ recovered after median sternotomy. An inspiratory pressure of 20cmH_2_O and PEEP of 8cmH_2_O resulted in a TV of 300 to 350mL. Arterial blood gas analysis at a fraction of inspired oxygen of 0.6 gave results of PaCO_2_ 41.8mmHg and PaO_2_ 197.5mmHg.

Resection of her tumor caused massive blood loss that required blood transfusion. During the operation, 3 to 5μg/kg/minute dopamine was given and her mean arterial pressure was kept above 60mmHg. Finally, her tumor was removed completely and had a weight of 10.5kg. On histological examination, the tumor was diagnosed as a mature teratoma without malignancy. Her total blood loss was 9790mL and 22 units of red cell concentrate, 19 units of fresh frozen plasma, and 20 units of platelet concentrate were transfused. After removal of her tumor, the same TV was maintained with lower inspiratory pressures and PEEP to prevent re-expansion pulmonary edema (RPE). On completion of the operation, she was transferred to the intensive care unit (ICU) and ventilated with an inspiratory pressure of 18cmH_2_O and PEEP of 8cmH_2_O, which resulted in a TV of 350mL. After her spontaneous respiration recovered, she was ventilated with pressure support and PEEP to maintain a TV of 300 to 350mL. She was extubated on postoperative day (POD) 2 and discharged from the ICU without any complication on POD 5.

## Discussion

Cases of GTS may present with a giant abdominal tumor [2,3] and general anesthesia and anesthetic management for tumor resection in these cases have several associated risks [4,5]. Circulatory and respiratory management are particularly difficult. The main concerns in anesthetic management are: (1) the influence on respiration and circulation of tumor pressure on the great vessels and lungs, (2) a risk of aspiration at intubation, (3) a risk of massive bleeding, and (4) occurrence of RPE. Due to these risks, it has been recommended that the tumor mass is reduced preoperatively to prevent circulatory and respiratory depression [4,5]. However, these cases involved cystic type tumors from which cystic fluid could be drained slowly. By contrast, our case had a solid mass tumor that could not be drained preoperatively. A further problem with giant abdominal tumors is the ventilatory management after administration of a muscle relaxant due to decreased lung and thoracic compliance caused by relaxation of the diaphragm and the enlarged abdomen, and the possibility of high airway pressures causing lung injury [6]. Therefore, it has been suggested that spontaneous ventilation should be maintained for as long as possible and that the inspiratory pressure should be kept under 20cmH_2_0 even if muscle relaxants are used [6,7]. In this case, use of a muscle relaxant was required to perform an abdominal section, and we chose to use the muscle relaxant just before the start of the operation and to perform awake intubation. We also decided to keep the inspiratory pressure under 20cmH_2_0 and to prepare for PCPS in a situation of worsening hypoxia, while seeking to prevent lung injury from high airway pressures. As expected, spontaneous respiration disappeared after administration of the muscle relaxant and we were unable to obtain a sufficient TV with positive pressure ventilation. An abdominal midline incision was performed and TV improved immediately. Overall, our strategy was to maintain spontaneous respiration for as long as possible and we recommend this approach in patients with a giant abdominal tumor that cannot be reduced in mass preoperatively.

Cases with GTS involve a giant mass that is sometimes very close to great vessels, but complete surgical resection is required to prevent recurrence [8,9]. Therefore, there is a risk of a large amount of bleeding. In our case, there was huge blood loss with resection of the tumor and extensive transfusion was required. However, we prepared for blood loss and cardiac failure preoperatively by cannulation and preparation for PCPS, and we were able to maintain stable hemodynamics throughout the surgery.

Finally, there was a risk of RPE after removal of the tumor. RPE develops upon rapid expansion of chronically collapsed lungs through a mechanism of increased pulmonary vascular permeability [10,11], with the duration of collapse of the lungs as one of the risk factors [12]. The onset of RPE is immediate and can often occur within an hour [13]. There is no standard method to prevent RPE, but in one case maintenance of a TV of 6 to 8mL/kg during surgery was thought to have caused RPE [7]. It has also been suggested that it is better to re-expand collapsed lungs very slowly with spontaneous respiration [14] and not to perform a lung recruitment maneuver to prevent RPE [7]. In our case, the lungs had been collapsed by the giant tumor for a long time and the patient had poor pulmonary function preoperatively. Therefore, she was a high risk case for RPE and there was possibility of development of RPE during surgery. To prevent this occurrence, we chose to re-expand the collapsed lungs very slowly and we maintained a relatively low TV after removal of the tumor, similar to that during spontaneous respiration preoperatively. Using this approach, we were able to manage the patient uneventfully during and after the operation.

## Conclusions

In summary, we have described a procedure for successful anesthetic management in a case of GTS with a giant abdominal tumor. Induction and maintenance of anesthesia had several associated risks and circulatory management required extensive transfusion. Respiratory management was achieved by avoiding use of a muscle relaxant before the operation to maintain spontaneous respiration and by maintaining a relatively low TV, similar to that during spontaneous respiration preoperatively, after removal of the tumor to prevent RPE.

## Consent

Written informed consent was obtained from the patient for publication of this case report and any accompanying images. A copy of the written consent is available for review by the Editor-in-Chief of this journal.

## Abbreviations

GTS: Growing teratoma syndrome; ICU: Intensive care unit; PaCO2: Partial pressure of carbon dioxide; PaO2: Partial pressure of oxygen; PCPS: Percutaneous cardiopulmonary support; PEEP: Positive end-expiratory pressure; POD: Postoperative day; RPE: Re-expansion pulmonary edema; SpO2: Pulse oximeter oxygen saturation; TV: Tidal volume; VC: Vital capacity.

## Competing interests

The authors declare that they have no competing interests.

## Authors’ contributions

NO was the major contributor in writing the manuscript. HIm and TT supervised the treatment of our patient and collected case data. HIs and HB revised the manuscript. All authors approved the final version of the manuscript.
